# Fall Detection with the Spatial-Temporal Correlation Encoded by a Sequence-to-Sequence Denoised GAN

**DOI:** 10.3390/s22114194

**Published:** 2022-05-31

**Authors:** Wei-Wen Hsu, Jing-Ming Guo, Chien-Yu Chen, Yao-Chung Chang

**Affiliations:** 1 Department of Computer Science and Information Engineering, National Taitung University, Tatung 950309, Taiwan; weiwenhsu@nttu.edu.tw (W.-W.H.); ycc@nttu.edu.tw (Y.-C.C.); 2 Department of Electrical Engineering, National Taiwan University of Science and Technology, Taipei 106335, Taiwan; m10707312@mail.ntust.edu.tw; 3 Advanced Intelligent Image and Vision Technology Research Center, National Taiwan University of Science and Technology, Taipei 106335, Taiwan

**Keywords:** fall detection, unsupervised learning, denoised GAN

## Abstract

Falling is a major cause of personal injury and accidental death worldwide, in particular for the elderly. For aged care, a falling alarm system is highly demanded so that medical aid can be obtained immediately when the fall accidents happen. Previous studies on fall detection lacked practical considerations to deal with real-world situations, including the camera’s mounting angle, lighting differences between day and night, and the privacy protection for users. In our experiments, IR-depth images and thermal images were used as the input source for fall detection; as a result, detailed facial information is not captured by the system for privacy reasons, and it is invariant to the lighting conditions. Due to the different occurrence rates between fall accidents and other normal activities, supervised learning approaches may suffer from the problem of data imbalance in the training phase. Accordingly, in this study, anomaly detection is performed using unsupervised learning approaches so that the models were trained only with the normal cases while the fall accident was defined as an anomaly event. The proposed system takes sequential frames as the inputs to predict future frames based on a GAN structure, and it provides (1) multi-subject detection, (2) real-time fall detection triggered by motion, (3) a solution to the situation that subjects were occluded after falling, and (4) a denoising scheme for depth images. The experimental results show that the proposed system achieves the state-of-the-art performance and copes with the real-world cases successfully.

## 1. Introduction

According to the reports from the World Health Organization (WHO), approximately 28–35% of the elderly fall each year, and it increases to 32–42% for those over 70 years of age [1]. Globally, accidents involving falls are the second leading cause of accidental deaths and are considered to be one of the most dangerous events for the elderly. Even if it did not cause personal injury, 47% of the elderly could not stand up by themselves after falling [2]. Based on the above reasons, a fall detection system that provides continuous monitoring is highly demanded so that medical aid can be applied immediately for the elderly after falling.

The studies about fall detection systems are mainly divided into two categories: fall detection based on wearable devices and situational awareness devices [3]. Accelerometers are commonly used in wearable devices, and a simple threshold can be set or derived by machine learning algorithms to define fall accidents [4]. The drawbacks of the approaches using wearable devices mainly came from the battery capacity of the device, and it is very likely that the user may forget to wear the device due to weak memory [5]. In addition, it is not suitable and comfortable for the elderly to wear electronic instruments [5,6,7]. On the other hand, for fall detection systems based on situational awareness devices, sensors, such as vibration sensors, microphones, cameras, etc., are deployed in the environment to detect falls. Yet, the sound or vibration signal may cause frequent false alarms due to the fall of objects. Consequently, the use of cameras with computer vision techniques have become the mainstream method for fall detection systems.

In previous studies, RGB cameras were frequently used as the input sensors for fall detection. Since RGB cameras are highly affected by the lighting conditions, the detection accuracy of the system is highly affected by the scene where the system is operated. In addition, fall accidents happen during the night very often when the elderly gets up from bed to use the restroom. Fall detection using an RGB camera fails to function without illumination. In addition, the elderly may frequently fall when using the restroom or taking a bath due to the slippery floor and using RGB images can lead to privacy violations. Accordingly, to tackle the problems mentioned above, the depth cameras have become a better choice of sensors for fall detection. The depth cameras obtain the depth information and achieve lighting independence through infrared structured light emission. As opposed to the RGB images, users’ detailed facial information is not recorded in the depth images and thus ensures users’ privacy. However, even though the depth images can be adopted to overcome the limitations of RGB images, it can be observed that the depth images are noisy and provide weak local gradient information of objects compared with RGB images [8], making it challenging for the deep learning models to detect objects in the depth images.

**Figure 1:**
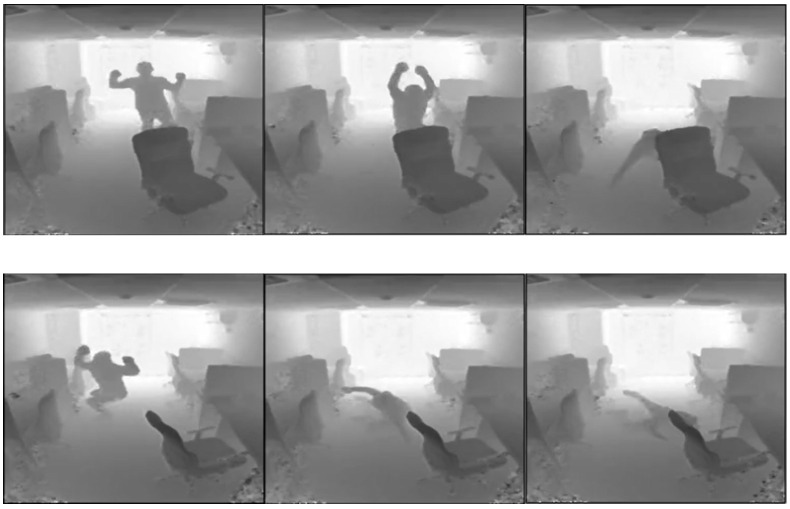
Two examples where the subjects were occluded by the furniture after falling.

For fall detection systems, compared with the normal activities of daily living (ADLs), the occurrence rate of fall accidents is relatively low. From the perspective of machine learning, data imbalance between positive samples (video clips with fall accidents) and negative samples (video clips with ADLs) is not conducive to the approaches of supervised learning classification in fall detection. In addition, the fall detection systems are expected to work in the real-world scenarios that the subjects are occluded after falling since the subjects are usually surrounded by beds, chairs, tables, and other furniture in the nursing home, as shown in Figure 1. According to the abovementioned reasons, in this study, we propose a framework using an unsupervised learning approach that is only trained with samples from normal activities of daily living and defined the fall accident as an abnormal event. The sequence-to-sequence Generative Adversarial Network (GAN) structure with the denoising scheme is designed to predict the future frames and learn the spatiotemporal changes in motion, instead of body postures. The main contributions of this study are summarized as follows.

(1) A sequence-to-sequence denoised GAN structure is proposed for fall detection, which achieves state-of-the-art performance.
(2) Our approach can perform multi-person fall detection.
(3) A denoising scheme is designed for depth image inputs.
(4) The proposed system can perform real-time fall detection in the real-world situation that subjects were occluded by the furniture after falling.

## 2. Related Work

With the recent advances in convolutional neural networks, several deep learning-based frameworks have been proposed for fall detection. Núñez-Marcos et al. [9] presented a framework that uses the optical-flow methods for action sequences analysis. However, their proposed framework is very sensitive to the lighting conditions, which also has a great influence on the optical flow field due to the RGB images they used as the inputs. Abobakr et al. [8] adopted ResNet to perform feature extraction on colorized depth frames. Subsequently, the extracted feature vector from ResNet became the input of two LSTM [10] layers to learn the changing characteristics among *T* consecutive frames. Finally, fully connected layers took the outputs from LSTM to classify if the subject was falling or not. Even though the depth images were used as the inputs to achieve lighting independence, the detection performance of their method is still greatly affected by the noises in the depth images. In addition, the problem of uneven collection of positive and negative samples still exists with their approach.

Tsai et al. [11] proposed a method that uses Kinect v2 to obtain the foreground depth image, and each pixel in the image represents the closest depth value. For subject detection, seven skeleton points were extracted through the operations of connecting components, morphology, and thinning. Subsequently, they trained a fall detection model based on the AlexNet [12] with five convolutional layers and two fully connected layers but took a 1-D feature vector from the flattened skeleton points, performing 1-D convolution. Even though the authors presented a lightweight model that can be embedded into the devices easily, their proposed framework can hardly be applied practically. Since their system can only detect people within 0.6 m from the camera, which is mounted horizontally, such approach is not sufficient to work on the practical scenarios for fall detection applications, not to mention the situations when the subjects are occluded by the furniture after falling or the scenario of multi-person fall detection.

Nogas et al. [13] proposed a method which defines the fall accident as an anomalous event and uses an autoencoder [14] to reconstruct the continuous depth frames. As a result, if the abnormal frames that involve fall accidents are fed into the autoencoder model for reconstruction, the fall alarm is triggered when the autoencoder model fails to reconstruct those anomalous frames back to their corresponding input frames precisely since the characteristics of the motion from those frames were not learned by the trained model in the training process. Accordingly, their proposed system is lighting invariant that can perform continuous monitoring, and the problem of imbalanced class training data can be avoided with the unsupervised learning approach. However, their denoising scheme is merely adequate for the inputs of depth images, so that the post-processing and the statistical information are needed for their approach to boost the detection performance, becoming inadequate to perform real-time fall detection.

## 3. Proposed Methods

**Figure 2:**
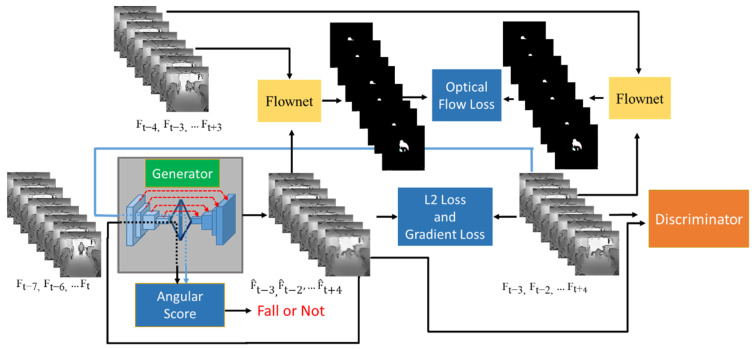
Overall architecture. The model takes the sequences of *N* consecutive frames and perform *N*/2 future frame predictions. If the trained model fails to reconstruct the future frames well, it means an anomalous event just happened, and the system will trigger the alarm. In our experiments, *N* was set at eight, considering the execution time for real-time detection and the limitation of GPU memory.

In this study, IR-depth images and thermal images are used as the input source for fall detection to reduce the influence of lighting conditions and avoid privacy violations. In addition, only the frame sequences with normal ADLs are collected in the training dataset for frame reconstruction, so that the frame sequences with fall accidents will lead to bad reconstruction by the trained model and trigger the anomaly alarm. To achieve that, a model with generative adversarial network (GAN) structure that takes frame sequences to capture the properties of spatiotemporal changes in motion and produces the corresponding reconstruction for future frame predictions is proposed. Figure 2 illustrates the overall architecture. It is composed of the generator network, discriminator network, FlowNet [15], and the proposed denoising scheme for anomalous scoring. In the training phase, sequences of *N* consecutive frames are fed into the generator with the corresponding *N* consecutive frames as the ground truth, of which half of the frames come from the inputs, and another half of the frames are obtained from the following frames after input frames to learn the spatiotemporal features and perform future frame predictions in the inference phase.

### 3.1. Materials and Data Preprocessing

The proposed fall detection system takes IR-depth images and thermal images as inputs for the purpose of achieving lighting-invariant and privacy protection. For performance evaluation, two public datasets were used in the experiments: Thermal Fall Dataset [16] and UR Fall Detection Dataset [17]. The details of these two datasets are introduced as follows.

**Thermal Fall Dataset** **:**

**Figure 3:**
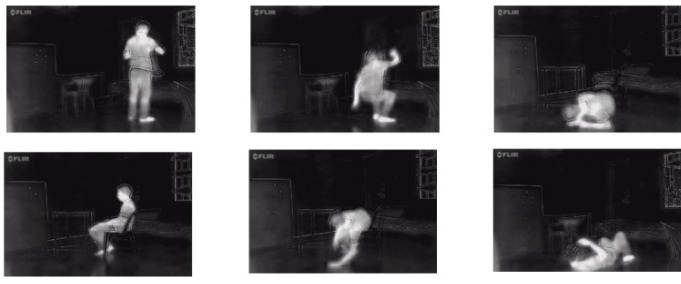
Samples from the Thermal Fall Dataset [16].

Thermal Fall Dataset [16] consists of several videos recorded by a FLIR ONE camera mounted on an Android phone that was installed parallell to the floor with the maxima measuring distance of 3 m. The frame rates of these videos are either 25 or 15 fps. There are 44 video clips, of which 35 clips include fall accidents and daily activities, and the remaining nine clips only contain daily activities. Additionally, the recorded video clips of falling and daily living activities were performed by 17 volunteer subjects. The spatial resolution of the thermal images is 640 × 480. Figure 3 shows some thermal image samples from the dataset.

**UR Fall Detection Dataset:**

**Figure 4:**
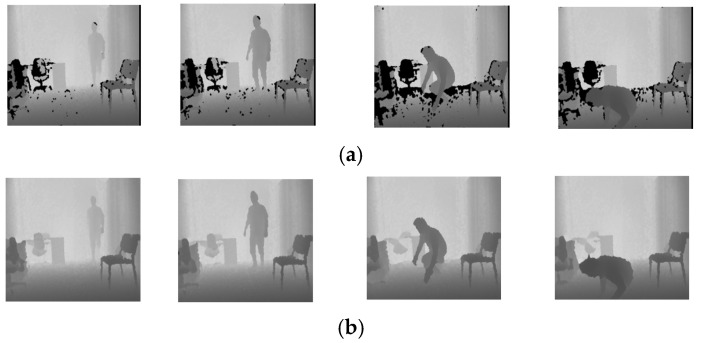
Samples from the URFD Dataset [17]. (**a**) Before hole filling. (**b**) After hole filling.

The UR Fall Detection Dataset [17] contains 70 depth videos captured by the Microsoft Kinect cameras that were mounted horizontally to the floor with the maxima distance of 3 m to the subjects. The frame rate is 30 fps, and the spatial resolution of each depth frame is 640 × 480. In total 30 videos are in the dataset with fall accidents and other daily activities, such as walking, sitting, squatting, and lying on beds. Those videos with fall accidents were recorded by five subjects who simulated two types of falling scenarios, including direct falls and falls from a chair. However, some missing pixels exist in depth images, caused by occlusion or scattering, multiple reflections, and transparent objects from the structured light sensors [18]. Those undefined pixels become noise that interfere with the deep learning models in feature extraction. Consequently, the preprocessing of the hole filling algorithm [19] is applied to reduce the noise. The comparison of input frames before and after applying the hole filling algorithm is demonstrated in Figure 4.

### 3.2. Network

In the proposed framework, the continuous motion features are extracted, and then used for future frame reconstruction by the generator. Meanwhile, the discriminator network is used to distinguish the reconstructed frames from the authentic frames. Moreover, to further reduce the influence of the noises from depth images, the reconstructed frames are sent back to the generator network to obtain the encoded features for comparison with the corresponding authentic frames, checking if any anomaly occurs in the testing phase. The design details are elaborated as follows.

#### 3.2.1. Generator

**Figure 5:**
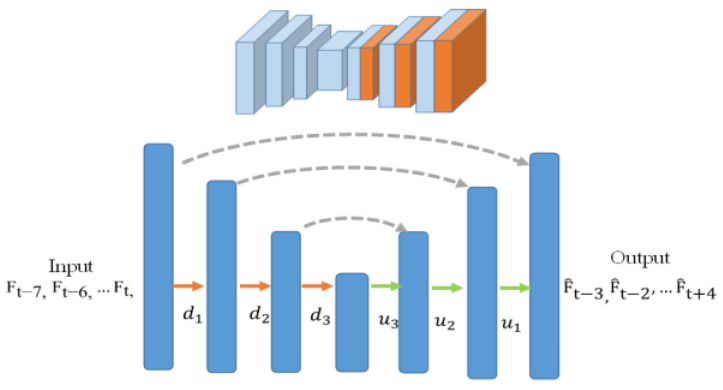
Generator network based on an encoder–decoder structure.

The generator network is based on an encoder–decoder structure [20] that takes frame sequences as the inputs to learn the spatiotemporal correlation of subjects’ motion in the training phase. In our experiments, sequences of eight consecutive frames are fed into the generator as the inputs. That is, the network takes the information from the previous 4 frames to predict future 4 frames. If the number of frames in an input sequence is less than 8, the information from the previous frames may not be sufficient to predict the future frames well. However, if we have more frames in the input sequence, the computing burden will increase, which is unfavorable to the system for performing real-time detection. The ground truth frames to learn are the corresponding eight consecutive frames with four frames shifted over the timeline to perform future frame predictions. In the testing phase, as is shown in Figure 5, a sequence of eight consecutive frames of depth images is fed into the trained model that produces the reconstructed sequence with four predicted future frames, which are ${\hat{F}}_{t+1}$, ${\hat{F}}_{t+2}$, ${\hat{F}}_{t+3}$, and ${\hat{F}}_{t+4}$.

#### 3.2.2. Discriminator

For the discriminator network, we followed the structure and settings of PatchGAN [21,22,23]. Since the problem of noises may still exist after hole filling, the concept of PatchGAN is adopted to divide the input depth image into *N* by *N* patches for judgments. (*N* = 70 in our experiments). After each block is judged, the ensemble scheme is applied to average the prediction results for classification. For the generated images, the high-frequency parts were frequently checked by the discriminator. As a result, it further reduces the influence of the noises in each small patch from the depth images.

#### 3.2.3. The Scoring Scheme Using Encoded Feature Comparison

**Figure 6:**
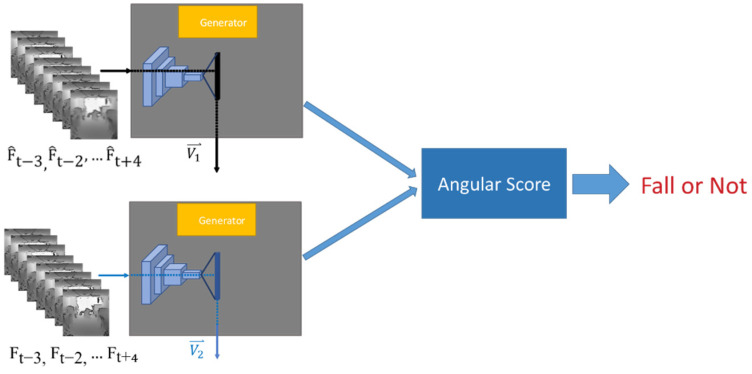
Scoring scheme using encoded feature comparison for anomaly detection.

Other than the hole-filling algorithm and PatchGAN architecture, the encoded feature comparison is also designed for denoising when using depth images as the inputs. Since only the main components from the dataset are encoded as features when training the generator model, the jittering noises in the depth images are not the stable components to be encoded and would be ignored. As is shown in Figure 6, the future frame predictions are fed back to the generator to obtain the encoded feature, $\vec{V_{1}}$, and compared with the encoded feature of the corresponding authentic frame sequence, $\vec{V_{2}}$. Consequently, it reduces the influence of jittering noises in the depth images. Accordingly, the discrepancy between two sequences can be measured by computing the included angle θ between their corresponding encoded feature vectors through Equation (1).

(1) $$\theta =\cos^{-1}\left(\frac{\vec{V_{1}}\cdot \vec{V_{2}}}{\left\|\vec{V_{1}}\|\times \|\vec{V_{2}}\right\|}\right)$$

As a result, if the input sequence contains the continuous frames with the motions of normal activities, the trained generator can reconstruct the corresponding future frames more precisely because the motions of normal activities have been learned from the training dataset and are predictable. Subsequently, the encoded feature vector $\vec{V_{1}}$ from the reconstructed sequence and the encoded feature vector $\vec{V_{2}}$ from the correspondingly authentic sequence are similar so that the included angle θ between them is small. Conversely, when the input sequence includes the continuous frames with the motions of fall accidents that are excluded in the training dataset, the generator will fail to predict the future motion based on the previous motion in the input sequence. Consequently, the included angle between the encoded feature vectors $\vec{V_{1}}$ and $\vec{V_{2}}$ becomes larger because the reconstructed frames are very different from the correspondingly authentic frames. Thus, the proposed scheme can be adopted to determine if a fall accident occurs in the input sequence.

### 3.3. Training Loss Functions

**Figure 7:**
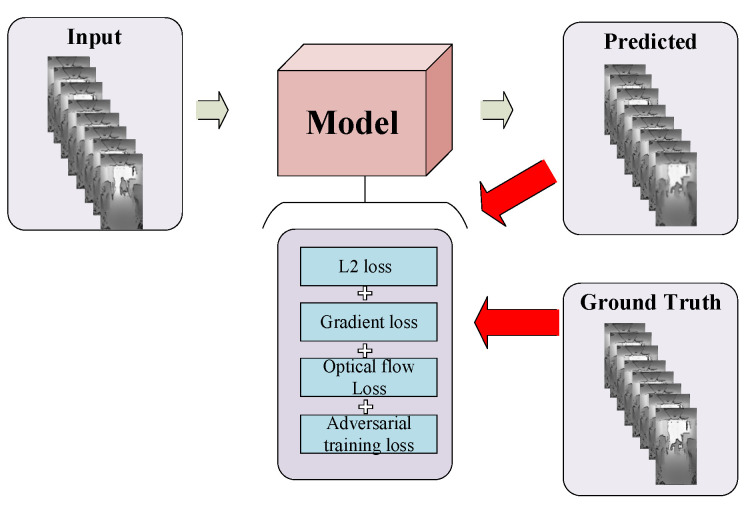
The L2 loss, gradient loss, optical-flow loss, and adversarial training loss are considered in the training phase for network optimization.

The proposed framework based on the generative adversarial network (GAN) structure comprises the generator network, the discriminator network, and the FlowNet. In training, the hybrid loss function that takes L2 loss, gradient loss, optical-flow loss, and adversarial training loss into consideration is designed for network optimization, as is shown in Figure 7.

#### 3.3.1. L2 Loss

For most of the image reconstruction tasks, $L_{2}$ loss is one of the most frequently adopted loss functions that directly computes the mean square error between the input and output images. The $L_{2}$ loss function is expressed as

(2) $$L_{2}\left(P\right)=\frac{1}{N}\sum_{p\epsilon P}{\left(y_{p}-pred\left(p\right)\right)}^{2},$$

where *p* represents the pixel in the image *P*, and *N* is the total number of pixels in the image. In Equation (2), *y_p_* stands for the pixel value of *p* from the target image, and *pred*(*p*) is the predicted pixel value of *p* from the reconstructed image.

However, compared with the frames in the input sequence, many high-frequency details are missing in the reconstructed frames. To tackle this problem, the loss function that considers the image gradient differences is needed in the optimization to enhance the detailed contours in the reconstructed results.

#### 3.3.2. Gradient Loss

To enhance the high-frequency details in the reconstructed frames, the gradient differences between the reconstructed results and the corresponding ground truth are taken into consideration in training. The mathematical expression of the gradient loss is as

(3) $$L_{gd}\left(\hat{Y\;},Y\right)=\sum_{i,j}(\left||Y_{i,j}-Y_{i-1,j}|-\left|{\hat{Y}}_{i,j}-{\hat{Y}}_{i-1,j}\right|\right|+\left||Y_{i,j-1}-Y_{i,j}|-\left|{\hat{Y}}_{i,j-1}-{\hat{Y}}_{i,j}\right|\right|)$$

where *i* and *j* stand for the spatial position of a pixel in the depth image. The variable $\hat{Y}$ represents the predicted frame, and *Y* is its corresponding ground truth. Herein, only the gradient intensity differences between two adjacent pixels are considered in the gradient loss. The adoption of gradient loss is expected to retain more high-frequency details so that the information of subjects in the depth image can be well captured.

#### 3.3.3. Optical-Flow Loss

The L2 loss and gradient loss mainly focus on the spatial characteristics of the differences in pixel intensity between the predicted frames and their corresponding ground truth. To capture the temporal features of frame-to-frame motion changes for future frame prediction, the FlowNet [15] is embedded into the framework to compute the changes in the optical-flow field from the reconstructed frames and the actual frames. The optical-flow loss is expressed as Equation (4), where FlowNet is denoted as F, $\hat{I}$ represents the predicted frame, and *I* is the corresponding ground truth.

(4) $$L_{\mathrm{op}}=\|F\left({\hat{I}}_{t+1},I_{t}\right)-F\left(I_{t+1},I_{t}\right)\|$$

#### 3.3.4. Adversarial Training Loss

The proposed framework is designed based on a GAN structure, containing a discriminator network and a generator network. The purpose of training discriminator networks is to distinguish the predicted 2*k*-frame sequence (${\hat{F}}_{t-\;k+1}$, …, ${\hat{F}}_{t}$, …, ${\hat{F}}_{t+k}$) from its correspondingly authentic sequence, i.e., ($F_{t-\;k+1}$, …, $F_{t}$, …, $F_{t+k}$). The label 1 represents the real depth images from the dataset, while the label 0 represents the reconstructed frames from the generator. For training a discriminator, the parameters of the network are optimized by minimizing the loss function, as expressed in Equation (5).

(5) $$L_{adv}^{D}\left(\hat{F},F\right)=\sum_{f=t-k+1}^{t+k}\sum_{i,j}\frac{1}{2}L_{MSE}\left(D{\left(F_{f}\right)}_{i,j},1\right)+\sum_{f=t-k+1}^{t+k}\sum_{i,j}\frac{1}{2}L_{MSE}\left(D{\left({\hat{F}}_{f}\right)}_{i,j},0\right)$$

On the other hand, the purpose of training generator networks is to reconstruct the frame sequence ${\hat{F}}_{f}$[*f* = *t* − *k* + 1:*t* + *k*] that confuses the discriminator network to classify the reconstructed sequence ${\hat{F}}_{f}$ as 1. Subsequently, for training a generator, the parameters of the network are optimized by minimizing the loss function, as expressed in Equation (6).

(6) $$L_{adv}^{G}\left(\hat{F}\right)=\sum_{f=t-k+1}^{t+k}\sum_{i,j}\frac{1}{2}L_{MSE}\left(D{\left({\hat{F}}_{f}\right)}_{i,j},1\right)$$

### 3.4. Training Details

All experiments were run on a *GTX2080 Ti* GPU and programmed with *TensorFlow*. For training and testing data, the size of the images was fixed at 256 × 256, and all pixels were rescaled to [–1, 1]. In the training phase, the batch size was set to 7 sequences (8 frames in a sequence) for an iteration due to the limitation of memory capacity from GPU, and Adam [24] was chosen as the optimizer. By our empirical settings, the initial learning rate was set at 0.0001, and it decays to one tenth every 70,000 iterations till it converges or terminates.

## 4. Experimental Results and Discussion

In this section, the public datasets, the thermal dataset [16] and the URFD dataset [17], were used for performance evaluation and the comparison with the previously proposed fall detection methods. In the experiments, the evaluation metrics of the Area Under the ROC Curve (AUC) and overall accuracy were computed for the assessment.

### 4.1. The Effect of the Combinations of the Loss Functions

**Figure 8:**
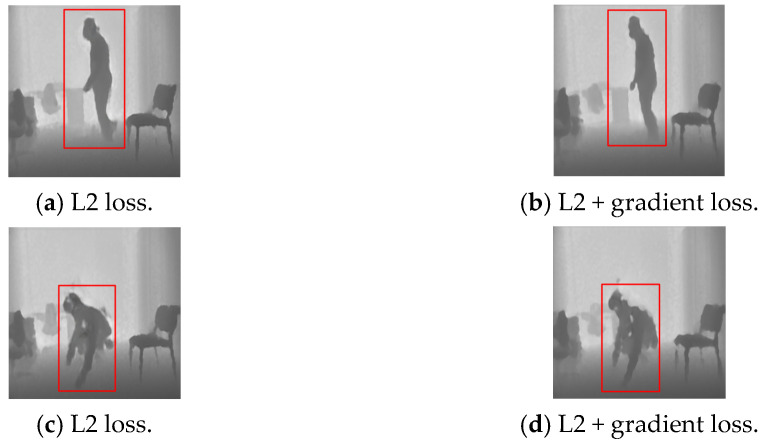
Comparisons of the reconstructed results between frames. (**a**) L2 loss only on normal activities. (**b**) L2 + gradient loss on normal activities. (**c**) L2 loss only on fall accidents. (**d**) L2 + gradient loss on fall accidents.

For network optimization, our goal is to search for the optimal loss function combination to boost the performance. In the beginning, L2 loss was applied to minimize the differences between the predicted frame and the corresponding authentic frame for optimization. However, many high-frequency details in the reconstructed images were missing when using the L2 loss only. Thus, the gradient loss was added to further enhance the reconstruction results. The reconstructed frames with a standing subject by “L2 loss” and “L2 + Gradient loss” are demonstrated in Figure 8a and Figure 8b, respectively. Compared with the reconstructed results by L2 loss only, the contours of the foreground subject are more precise and clearer as can be observed for the parts near the hand and ankle in Figure 8b when the model was trained with “L2 + Gradient loss” for the cases of normal activities. Conversely, for the reconstruction of frames in which fall accidents happen, the model trained with “L2 + Gradient loss” may fail to reconstruct those frames well, as shown in Figure 8d, compared with the results of the model trained with the L2 loss only, as shown in Figure 8c. That is because the cases with fall motion in testing were unseen by the models, and it is more sensitive to the edge contours for the model with the gradient loss involved, compared to the model trained with L2 loss only. It is favorable for the anomaly detection system because the better reconstruction on the normal cases leads to low false alarms while the poorer reconstruction on the anomalous cases can trigger the alarm more sensitively.

**Figure 9:**
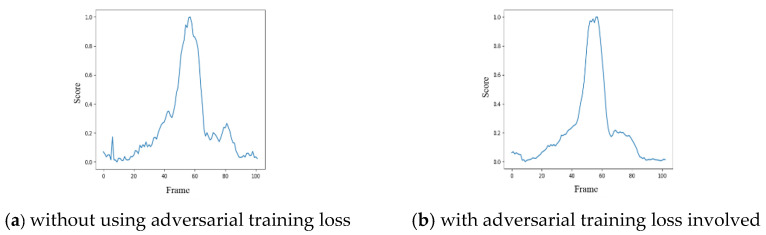
Influence of the jittering effect among frames (**a**) before and (**b**) after the denoising scheme of PatchGAN.

In addition, the unpredictable noises after hole filling lead to the jittering effect among frames, affecting the prediction results with respect to the inputs/outputs of frame sequences. In Figure 9, the subplot Figure 9a shows the influence of the jittering effect by the model trained with “L2 + gradient loss”, and the subplot Figure 9b is the scenario with the adversarial training loss involved, i.e., “L2 + gradient loss + adversarial training loss”. From observations, the PatchGAN structure in the proposed framework can smoothen the waves of the angular scores and reduce the influence of the jittering effect among frames. For a fall detection system, it can be adopted to reduce the number of false alarms effectively and make the system more robust in prediction.

### 4.2. Comparison with the Framework of DeepFall

In our proposed framework, the reconstruction error increases when an anomalous fall event occurs since the model cannot correctly reconstruct the abnormal frames and triggers the alarm for fall detection. However, frame-by-frame analysis for determining whether the reconstruction error becomes higher may cause false alarms easily [13]. Consequently, the statistical approaches as the post-processing were adopted in [13] for further false alarm reduction, yet cannot be performed in real-time detection. In contrast, the denoising scheme of encoded feature comparison proposed in this paper reduces the influence of jittering noises on the reconstruction results successfully and maintains the system running in real-time. Table 1 lists the performance of different approaches with the metric of AUC for comparison.

In Table 1, DAE and CAE stand for the implementations of fall detection systems using the structures of Deep AutoEncoder and Convolutional AutoEncoder, respectively. The DSTCAE-C3D *Cσ* and *Cµ* are the approaches with their proposed anomaly scoring scheme in [13] that incorporates the statistical information from the past and future as the post-preprocessing. As it can be observed in Table 1, without the post-processing, our proposed structure, i.e., S2SdGAN (Sequence-to-Sequence denoised GAN), significantly outperforms the models of DAE, CAE-UpSampling, and CAE-Deconv in [13]. Our proposed method without post-processing achieves the same level of detection performance as the scenarios of DSTCAE-C3D *Cσ* and *Cµ* in [13] that need post-processing. If we rescale our anomaly scores to the range of 0–1 as the post-processing to perform the off-line version of our proposed system, namely S2SdGAN-normalized, in Table 1, the performance surpasses all the compared methods. In our experiment, the threshold of the angular score that triggers the anomaly alarm was determined by grid search for the value that achieved the overall best performance in the training dataset. However, the best thresholds for the anomalous score are different case-by-case due to the different subjects and their motions. Therefore, the off-line version that performs the normalization of the anomalous scores for each video clip in [13] can strike better performance and robust fall predictions.

In addition, the score gaps Δ_s_ were computed to validate if the two classes (falls and normal ADLs) are well-separated in the systems. The larger score gap indicates the greater difference between two classes, i.e., “fall” and “non-fall”, that can be well-distinguished by the systems. As a result, the system with a larger score gap can achieve better accuracy in detection, leading to a lower false alarm rate. The score gap Δ_s_ is computed, followed by Equation (7).

(7) $${∆}_{s}=\frac{1}{N1}\sum \mathrm{Score}\left(\hat{F},1\right)-\frac{1}{N0}\sum \mathrm{Score}\left(\hat{F},0\right)$$

In Equation (7), $\hat{F}$ represents the reconstructed frame from the network, and Score ($\hat{F}$, 1) are the anomalous scores derived by the fall alarm system for those frames labeled as “fall/1” in the ground truth. *N*1 and *N*0 are the total number of frames defined as “fall/1” and “non-fall/0”, respectively. The comparison of performance with score gap Δ_s_ and AUC between DeepFall-DSTCAE-C3D [13] and our proposed method (S2SdGAN-normalized) is listed in Table 2. The score gaps by our proposed method are higher than the score gaps by the approach in [13] on both datasets, and it implies that a higher detection rate and a lower false alarm rate can be obtained, leading to a higher AUC.

**Figure 10:**
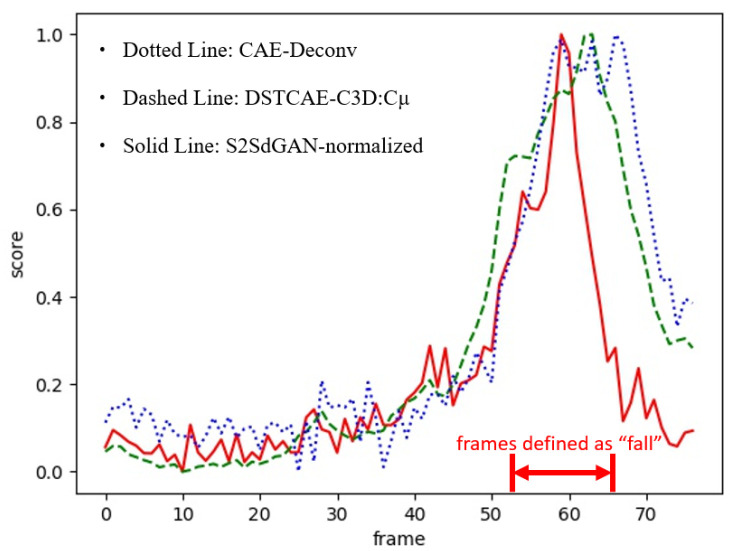
Comparison of the normalized anomalous scores corresponding to the frames of “fall” and “non-fall” among different approaches.

The better detection performance achieved by our proposed framework should contribute to our denoising schemes of applying the PatchGAN-like structure and the encoded feature comparison. Without a proper denoising scheme to tackle the noise problem in the depth images, the anomaly detection system cannot distinguish whether the poor reconstruction is caused by the abnormal motion from the foreground subject or just the unpredictable noise from the background, arousing higher false-positive rates in detection. Figure 10 shows the anomalous scores corresponding to the frames of “fall” and “non-fall” using different approaches on a sample from the UR Fall Detection dataset. As it can be observed in Figure 10, our proposed method can keep most of the anomalous scores below the threshold value of 0.3 for the non-fall frames, leading to the lowest number of false-positive frames among others.

**Figure 11:**
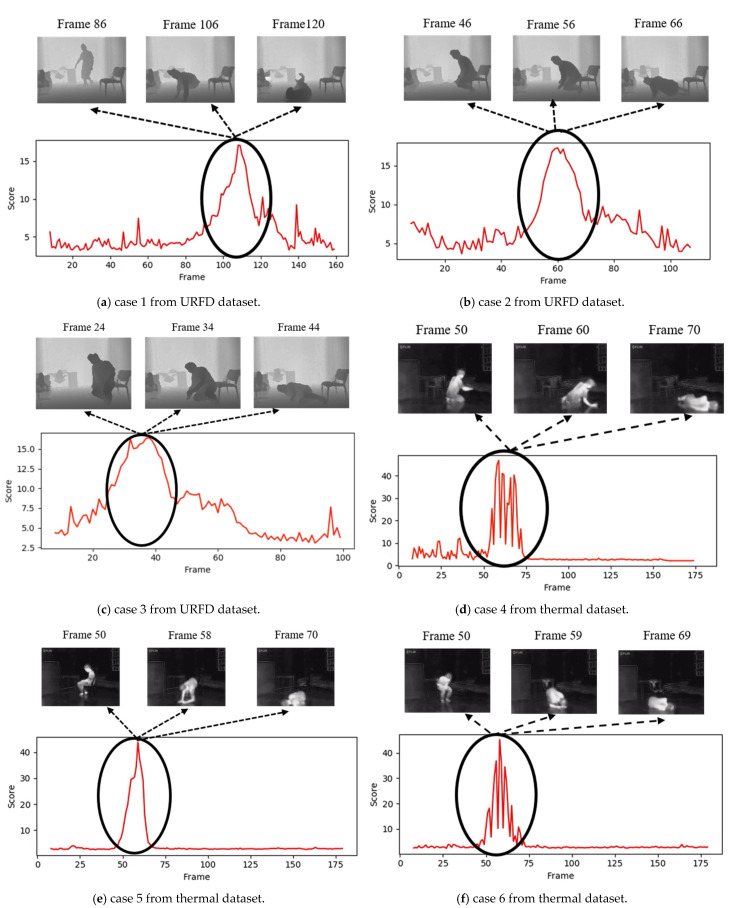
Six examples of the fall detection results from the UR Fall Detection (URFD) dataset (**a**–**c**), and thermal dataset (**d**–**f**).

The experimental results show better and more robust performance on both the UR Fall Detection dataset and the thermal dataset. Some examples of the fall detection results are shown in Figure 11, and it can be observed that the anomalous scores rise and drop following the subjects’ motion changes during falls. The results have proven that the proposed anomalous scoring scheme works successfully in fall detection.

### 4.3. Performance Comparison on the UR Fall Detection Dataset

The performance comparison among the previously proposed methods that conducted their experiments on the UR Fall Detection dataset is presented in Table 3. All experiments were performed through 5-fold cross-validation, and the metric of overall accuracy is used for evaluation. Even though our performance did not win the best overall accuracy among all, the proposed framework achieves the same detection level or even surpasses those approaches taking RGB images as inputs. Obviously, compared with using RGB images or RGB + depth images (RGB-D), it is much more challenging for the fall detection systems to take depth maps as inputs only since the blurry shape of subjects and noisy background interfere with the prediction results severely. Most importantly, our proposed fall detection system achieves users’ privacy protection and lighting-invariant, which can work on real-world situations.

### 4.4. Performance Assessment for the Real-World Situations

**Figure 12:**
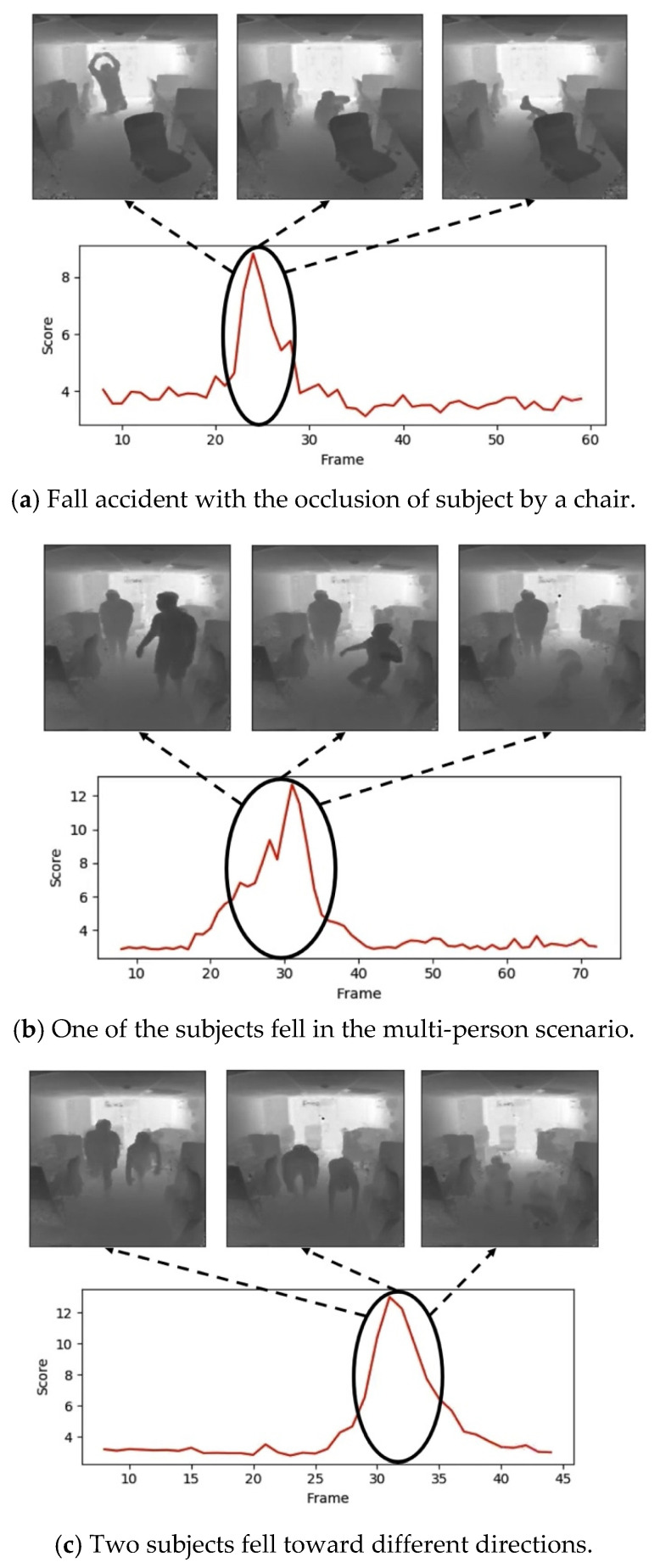
Fall detection for the scenarios of occlusion of subjects and multi-person scene to simulate the real-world situations.

Except for the above two public datasets used for performance comparison with the previously proposed frameworks, our designed fall detection system was further assessed to determine whether it can work on the real-world situations. For the practical fall detection systems, the cameras are usually mounted on the ceiling or walls with a certain angle to the subjects. Therefore, here we had our sensor of Time-of-Flight (ToF) mounted at 2.2 m height with a certain angle toward to the subjects and the maxima measuring distance of 4.5 m to simulate the real-world situations. The model trained on the UR Fall Dataset was used for inference since the sensor of ToF also generates the depth images as the inputs. Figure 12 shows the detection results with the scenarios of occlusion of subjects after falling and multi-person scenes to simulate the real-world situations. The results suggest that the proposed anomaly detection scheme is robust and transferable to tackle real-world problems.

In addition, in the inference phase, the model and the input sequence take about 3.7 GB in the GPU memory, and the execution frames-per-second is 12, tested under NVIDIA RTX 2080 Ti. Accordingly, to achieve real-time detection for the real-world applications, the input frame sequences are collected by sampling every 3rd frame, so the proposed fall detection system will check whether the subjects are falling every second.

## 5. Conclusions

In this study, a GAN-like network that inspects the reconstruction quality of future frame sequences based on the subject’s motion was proposed for fall detection. The proposed framework can cope with the extreme scenario when the subjects were blocked or unseen after falling. In addition, to ensure users’ privacy, the depth images or the thermal images are used as the inputs, and several denoising schemes are proposed to prevent the noises in the input images from interfering with the prediction results. Accordingly, the unsupervised learning approach is adopted that considers fall accidents as an anomalous event to tackle the problem of an imbalanced dataset from data acquisition. Except for the advantages mentioned above, the experimental results on two public datasets show that our proposed framework for fall detection achieves the state-of-the-art performance. For future work, a scheme to derive the adaptive threshold that performs device-invariant features for the system can be developed. In that case, the framework can work for various sensor devices without having separate threshold settings for each device.

## Figures and Tables

**Table 1:** Performance comparison between DeepFall [13] and our proposed S2SdGAN using the metric of AUC on two public datasets.

Method	Thermal	UR-Filled
DAE [13]	0.65	0.75
CAE-UpSampling [13]	0.73	0.67
CAE-Deconv [13]	0.75	0.76
DSTCAE-C3D:*Cσ* [13]	0.97	0.80
DSTCAE-C3D:*Cµ* [13]	0.93	0.86
S2SdGAN (our proposed)	0.952	0.872
S2SdGAN-normalized (our proposed off-line version)	**0.975**	**0.953**

**Table 2:** Performance comparison using the metrics of Score Gap (Δ_s_) and AUC on the datasets of THERMAL and UR-Filled.

	Thermal		UR-Filled	
	∆_s_	AUC	∆_s_	AUC
DeepFall-DSTCAE-C3D [13]	0.416	97%	0.395	86%
S2SdGAN-normalized	**0.670**	**97.5%**	**0.474**	**95.3%**

**Table 3:** Performance comparison among fall detection approaches on the UR Fall Detection dataset.

Method	Classifier	Input	Overall Accuracy
Kwolek et al. [17]	SVM	Depth maps only	90.00%
Goudelis et al. [25]	SVM	RGB-D	87.76%
Zerrouki et al. [26]	SVM + HMM	RGB	96.88%
Marcos et al. [9]	CNN	RGB	95.00%
S2SdGAN-normalized (the proposed)	GAN	Depth maps only	95.42%

## Data Availability

Not applicable.

## References

[1] World Health Organization (2015). World Report on Ageing and Health.

[2] Tinetti M.E., Liu W.-L., Claus E.B. (1993). Predictors and prognosis of inability to get up after falls among elderly persons. JAMA.

[3] Igual R., Medrano C., Plaza I. (2013). Challenges, issues and trends in fall detection systems. Biomed. Eng. Online.

[4] Wu G., Xue S. (2008). Portable preimpact fall detector with inertial sensors. IEEE Trans. Neural Syst. Rehabil. Eng..

[5] Abobakr A., Hossny M., Nahavandi S. (2017). A skeleton-free fall detection system from depth images using random decision forest. IEEE Syst. J..

[6] Demiris G., Rantz M.J., Aud M.A., Marek K.D., Tyrer H.W., Skubic M., Hussam A.A. (2004). Older adults’ attitudes towards and perceptions of ‘smart home’technologies: A pilot study. Med. Inform. Internet Med..

[7] Stone E.E., Skubic M. (2014). Fall detection in homes of older adults using the Microsoft Kinect. IEEE J. Biomed. Health Inform..

[8] Abobakr A., Hossny M., Abdelkader H., Nahavandi S. Rgb-d fall detection via deep residual convolutional lstm networks. Proceedings of the 2018 Digital Image Computing: Techniques and Applications (DICTA).

[9] Núñez-Marcos A., Azkune G., Arganda-Carreras I. (2017). Vision-based fall detection with convolutional neural networks. Wirel. Commun. Mob. Comput..

[10] Hochreiter S., Schmidhuber J. (1997). Long short-term memory. Neural Comput..

[11] Tsai T.-H., Hsu C.-W. (2019). Implementation of Fall Detection System Based on 3D Skeleton for Deep Learning Technique. IEEE Access.

[12] Krizhevsky A., Sutskever I., Hinton G.E. (2012). Imagenet classification with deep convolutional neural networks. Advances in Neural Information Processing Systems.

[13] Nogas J., Khan S.S., Mihailidis A. (2020). DeepFall: Non-Invasive Fall Detection with Deep Spatio-Temporal Convolutional Autoencoders. J. Healthc. Inform. Res..

[14] Hinton G.E., Salakhutdinov R.R. (2006). Reducing the dimensionality of data with neural networks. Science.

[15] Dosovitskiy A., Fischer P., Ilg E., Häusser P., Hazirbas C., Golkov V., van der Smagt P., Cremers D., Brox T. Flownet: Learning optical flow with convolutional networks. Proceedings of the IEEE International Conference on Computer Vision.

[16] Vadivelu S., Ganesan S., Murthy O.R., Dhall A. Thermal imaging based elderly fall detection. Proceedings of the Asian Conference on Computer Vision.

[17] Kwolek B., Kepski M. (2014). Human fall detection on embedded platform using depth maps and wireless accelerometer. Comput. Methods Programs Biomed..

[18] Shotton J., Fitzgibbon A., Cook M., Sharp T., Finocchio M., Moore R., Kipman A., Blake A. Real-time human pose recognition in parts from single depth images. Proceedings of the CVPR 2011.

[19] Bertalmio M., Bertozzi A.L., Sapiro G. Navier-stokes, fluid dynamics, and image and video inpainting. Proceedings of the 2001 IEEE Computer Society Conference on Computer Vision and Pattern Recognition.

[20] Ronneberger O., Fischer P., Brox T. U-net: Convolutional networks for biomedical image segmentation. Proceedings of the International Conference on Medical Image Computing and Computer-Assisted Intervention.

[21] Isola P., Zhu J.-Y., Zhou T., Efros A.A. Image-to-image translation with conditional adversarial networks. Proceedings of the IEEE Conference on Computer Vision and Pattern Recognition.

[22] Zhu J.-Y., Park T., Isola P., Efros A.A. Unpaired image-to-image translation using cycle-consistent adversarial networks. Proceedings of the IEEE International Conference on Computer Vision.

[23] Li C., Wand M. Precomputed real-time texture synthesis with markovian generative adversarial networks. Proceedings of the European Conference on Computer Vision.

[24] Kingma D.P., Ba J. (2014). Adam: A method for stochastic optimization. arXiv.

[25] Goudelis G., Tsatiris G., Karpouzis K., Kollias S. Fall detection using history triple features. Proceedings of the 8th ACM International Conference on PErvasive Technologies Related to Assistive Environments.

[26] Zerrouki N., Houacine A. (2018). Combined curvelets and hidden Markov models for human fall detection. Multimed. Tools Appl..

